# Evaluation of the PowerChek™ Respiratory Virus Panel 1/2/3/4 for the Detection of 16 Respiratory Viruses: A Comparative Study with the Allplex™ Respiratory Panel Assay 1/2/3 and BioFire^®^ Respiratory Panel 2.1 *plus*

**DOI:** 10.3390/diagnostics15212713

**Published:** 2025-10-27

**Authors:** Hyeongyu Lee, Rokeya Akter, Jong-Han Lee, Sook Won Ryu

**Affiliations:** 1 Department of Laboratory Medicine, Wonju College of Medicine, Yonsei University, Wonju 26426, Republic of Korea; sinoull@naver.com (H.L.); akterbinteha30@yonsei.ac.kr (R.A.); 2 Research Institute of Metabolism and Inflammation, Wonju College of Medicine, Yonsei University, Wonju 26426, Republic of Korea; 3 Department of Laboratory Medicine, School of Medicine, Kangwon National University, Chuncheon 24341, Republic of Korea

**Keywords:** molecular diagnostics, respiratory infections, respiratory viruses, multiplex RT-PCR, Kogene PowerChek™ RVP, Seegene Allplex™ RP, BioFire^®^ RP 2.1*plus*, performance evaluation

## Abstract

**Background/Objectives**: Acute respiratory infections remain a major global public health concern affecting individuals across all ages. Accurate and rapid diagnosis of respiratory pathogens is crucial for effective patient management and infection control. Multiplex real-time polymerase chain reaction (PCR) assays have gained prominence over conventional methods for routine viral detection in clinical laboratories owing to their enhanced sensitivity and specificity; however, comparative performance data for PowerChek™ RVP remain limited. This study aimed to evaluate the diagnostic performance of the PowerChek™ Respiratory Virus Panel 1/2/3/4, which detects 16 respiratory viruses, including SARS-CoV-2, in nasopharyngeal swab (NPS) specimens. **Methods**: Overall, 336 NPS specimens were analyzed using the PowerChek™ RVP, BioFire^®^ RP 2.1*plus*, and Allplex™ RP assays, with nucleic acid extraction performed using the Advansure™ E3 system. The performance metrics were calculated using two-by-two contingency tables. **Results**: Among 336 NPS specimens (232 positive, 104 negative), PowerChek™ RVP detected 226 positives with minimal discrepancies, showing high concordance with BioFire^®^ RP 2.1*plus* (accuracy 94.6%, kappa 0.843–1.000). Fifteen discordant cases were identified in this study. Eleven could not be sequenced because of amplification failure and most had high Ct values (>30). Sequencing of four samples confirmed concordance with BioFire^®^ RP 2.1*plus* and PowerChek™ RVP, whereas Allplex™ RP showed false-negative results. **Conclusions**: The PowerChek™ RVP assay demonstrated a high level of relative sensitivity, specificity, accuracy, diagnostic predictive values and strong concordance with comparable reference assays in identifying its targets. This assay is a reliable and efficient diagnostic tool for clinical laboratories to facilitate the accurate identification of respiratory pathogens.

## 1. Introduction

Acute respiratory infections (ARIs) represent a major global health burden that significantly contributes to morbidity and mortality worldwide [1]. According to the World Health Organization, infectious respiratory diseases are the leading cause of years of life lost due to death or disability [2]. ARIs are caused by bacterial and viral pathogens, with clinical symptoms ranging from fever and cough to wheezing, tachypnea, and respiratory distress. The treatment varies depending on the underlying causative agent [3].

Viruses account for approximately 80% of ARIs. Both influenza viruses (IFVs) and non-IFVs, including adenovirus (AdV), human bocavirus (HBoV), coronavirus (CoV), respiratory syncytial virus (RSV), human enterovirus (HEV), human rhinovirus (HRV), parainfluenza virus (PIV), and human metapneumovirus (hMPV), are common pathogens [4]. Accurate and timely detection of viral pathogens is essential for guiding clinical management, reducing inappropriate antibiotic use, and preventing unnecessary laboratory testing [5].

Traditionally, viral culture has been considered the gold standard for respiratory virus diagnosis. However, its long total turnaround time (TAT) and technical limitations restrict its routine use. Serological testing is widely used; however, its low sensitivity and specificity, together with the need for paired sera, limit its applicability in the clinical field [6]. Molecular diagnostic methods, particularly polymerase chain reaction (PCR)-based assays, have largely replaced traditional techniques. They offer higher sensitivity, specificity, and faster results [6,7]. Multiplex reverse transcription PCR (RT-PCR) allows the simultaneous detection of multiple respiratory pathogens in a single reaction, reduces TAT, conserves clinical specimens, and enhances diagnostic efficiency [8,9,10]. This method is now widely recommended in clinical guidelines, particularly for high-risk populations [11,12].

Several commercial multiplex PCR assays are available, among which the BioFire^®^ Respiratory 2.1 *plus* Panel (RP 2.1*plus*; BioMérieux, Salt Lake City, UT, USA) and the Allplex™ Respiratory Panel (RP) assays (Seegene Inc., Seoul, Republic of Korea) are widely validated and frequently used in clinical practice in the Republic of Korea. In contrast, the PowerChek™ Respiratory Virus Panel (RVP; Kogene Biotech, Seoul, Republic of Korea) has recently been introduced, and comparative performance data remain limited.

This study aimed to evaluate the diagnostic performance of the PowerChek™ RVP 1/2/3/4 in detecting respiratory viruses from nasopharyngeal swab specimens. Its performance was compared with the BioFire^®^ RP 2.1*plus* and Allplex™ RP 1/2/3 assays. The concordance and diagnostic accuracy were assessed using relative sensitivity, relative specificity, predictive values, and Cohen’s kappa coefficient.

## 2. Materials and Methods

### 2.1. Ethics Approval

This study was approved by the Institutional Review Board of the Kangwon National University Hospital (IRB Number: KNUH-2024-09-010). Residual clinical samples or nucleic acid samples obtained from patients who underwent routine diagnostic testing were utilized. These samples were unrelated to this clinical trial, and no personally identifiable information was collected.

### 2.2. Clinical Specimen

A total of 336 nasopharyngeal swabs (NPS) in viral transport medium (ASAN Transport Medium for Virus, *Chlamydia & Ureaplasma*; Asan Pharm, Dongdaemun-gu, Seoul, Republic of Korea) were collected from patients with suspected respiratory infections at Kangwon National University Hospital between 2023 and 2024. All samples were initially tested with the BioFire^®^ RP 2.1*plus* panel, and subsequently stored at −70 °C until further testing with the PowerChek™ RVP 1/2/3/4 and Allplex™ RP 1/2/3 assays.

**Figure 1:**
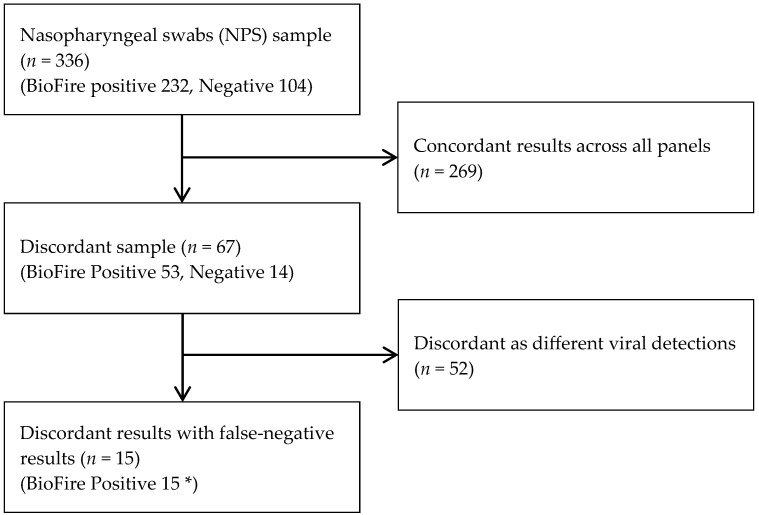
A total of 336 clinical samples were initially included. Samples were sequentially excluded due to concordant results across all panels or additional viral detections. After these exclusions, 15 discordant samples remained for analysis. * All 15 samples were subjected to viral sequencing.

Samples were excluded if the volume was <300 µL, storage conditions were inadequate, contamination was detected, or freeze–thaw cycles exceeded three. In case of discordant results, additional analyses were performed using viral sequencing. The overall selection process for the discordant samples included in the final analysis is summarized in Figure 1.

### 2.3. PowerChek™ RVP 1/2/3/4

The PowerChek™ RVP 1/2/3/4 is a KMFDS-approved in vitro diagnostic multiplex real-time RT-PCR assay. It detects 16 respiratory viruses, including AdV, CoVs (229E, NL63, and OC43), influenza A/B, HBoV, hMPV, HRV/HEV, RSV, PIV1–4, and SARS-CoV-2 [13]. Nucleic acids were extracted from 200 µL of the specimen using the Advansure™ E3 system (Invitros, Seoul, Republic of Korea). Amplification was performed on a CFX96™ Real-Time PCR System (Bio-Rad, Hercules, CA, USA) according to the manufacturer’s protocol.

A result was considered positive when the fluorescence signal crossed the threshold within the assay-specific cut-off value (Ct ≤ 33–34) during 40 amplification cycles, depending on each viral target. For SARS-CoV-2, both nucleocapsid (N) and open reading frame 1ab (ORF1ab) targets are required to confirm positivity. GAPDH was used as an internal control (IC) to monitor the quality of nucleic acid extraction and amplification.

### 2.4. Allplex™ RP 1/2/3

The Allplex™ Respiratory Panel 1/2/3 is a multiplex real-time RT-PCR assay for respiratory virus detection. It detects 19 respiratory viruses, including AdV, CoVs (229E, NL63, and OC43), influenza A (influenza A, H1, H1pdm09 and H3), influenza B, HBoV, hMPV, HRV, HEV, RSV A, RSV B, PIV1–4.

PCR were prepared and run on the CFX96™ Real-Time PCR System according to the manufacturer’s instructions. A sample was defined as positive if amplification occurred with a Ct value < 42 during 45 PCR cycles. The results were analyzed using the Seegene Viewer v2.0 software. The assay employs Seegene’s multiple detection temperature (MuDT™) technology, which enables simultaneous detection of multiple analytes in a single fluorescence channel without the need for melting curve analysis, with a TAT of approximately 210 min. Bacteriophage MS2 phage was used as an IC to verify the efficiency of nucleic acid extraction and amplification.

### 2.5. BioFire^®^ Respiratory Panel 2.1 plus (RP 2.1plus)

The BioFire^®^ RP 2.1*plus* is a fully automated sample-to-result system. It integrates nucleic acid extraction, reverse transcription, nested multiplex PCR, and automated detection. Testing was performed according to the manufacturer’s instructions. The results were automatically interpreted using the BioFire Software (TORCH System, version 3.2.4.0). The TAT was approximately 45 min per sample. The panel detected 19 respiratory viruses, including influenza A (H1N1, H3N2, and 2009-H1N1), influenza B, RSV, AdV, hMPV, HRV/HEV, four seasonal coronaviruses (229E, NL63, OC43, and HKU1), PIV1–4, MERS-CoV, and SARS-CoV-2. It also detected four atypical bacteria: *Bordetella pertussis*, *Bordetella parapertussis*, *Chlamydia pneumoniae*, and *Mycoplasma pneumoniae*. The BioFire^®^ RP 2.1*plus* Panel includes two ICs to ensure assay validity. The control material is carried through all stages of the test process, including lysis, nucleic acid purification, reverse transcription, PCR1, dilution, PCR2, and DNA melting. The RNA process control, derived from *Schizosaccharomyces pombe*, monitors all analytical steps from lysis to DNA melting. The PCR2 control, consisting of a synthetic DNA template in the array, verifies the PCR2 reaction. Both controls must be positive for a valid result.

### 2.6. Comparison of Three Real-Time PCR Methods for Respiratory Virus Detection

The comparative analysis of three commercially available real-time PCR platforms—PowerChek™ RVP, Allplex™ RP, and BioFire^®^ RP 2.1*plus*—is shown in Table 1. It is based on key performance parameters for the detection of respiratory viruses.

### 2.7. Further Study for Discordant Results

Additional analyses were performed to resolve the discrepancies among the three panels. For targets detected by the BioFire^®^ RP 2.1*plus* but not by the PowerChek™ RVP or Allplex™ RP, confirmatory testing was performed to verify the BioFire^®^ RP 2.1*plus* results, followed by viral sequencing. Further experiments were conducted on HRV/HEV, where BioFire^®^ RP 2.1*plus* showed negative results, but PowerChek™ RVP or Allplex™ RP yielded multiple positive results.

RNA extracted from BioFire^®^ RP 2.1*plus*-confirmed HRV/HEV-positive specimens and validated reference materials was serially diluted in nuclease-free water to assess and compare the analytical sensitivity of the Allplex™ RP and PowerChek™ RVP assays. Du-plicate qPCR were performed for each dilution. The reference materials used in the experiments were Coxsackievirus A6, strain Gdula (ATCC VR-1801; ATCC, Manassas, VA, USA) for HEV and HRV 14 (KBPV-VR-39D; Korea Bank for Pathogenic Viruses, Seoul, Republic of Korea).

### 2.8. Statistical Analysis

BioFire^®^ RP 2.1*Plus* was used as the reference standard to compare the performance of the PowerChek™ RVP and Allplex™ RP assays. When BioFire^®^ RP 2.1*Plus* produced a negative result but the other two panels were positive, the result was classified as a false positive. Conversely, when BioFire^®^ RP 2.1*Plus* detected a pathogen but the other panels were negative, the result was classified as a false negative. Using these definitions, the relative sensitivity was calculated to represent the ability of each assay to correctly identify positive cases compared to the reference.

Relative specificity reflects the ability to correctly identify negative cases, while relative accuracy indicates the overall agreement between each assay and the reference method. Sensitivity, specificity, accuracy, positive predictive value (PPV), negative predictive value (NPV), and Cohen’s kappa coefficient with 95% confidence intervals were calculated using two-by-two contingency tables. All statistical analyses were performed using Microsoft Excel (Microsoft Corporation, Redmond, WA, USA) and GraphPad QuickCalcs (https://www.graphpad.com/quickcalcs/ (accessed on 31 August 2025)) [14].

## 3. Results

### 3.1. Detection of 16 Respiratory Viruses

A total of 336 NPS specimens (224 positives and 104 negatives by BioFire^®^ RP 2.1*plus,* and 8 HBoV positives by Allplex™ RP) were analyzed to compare the performance of the PowerChek™ RVP 1/2/3/4 and Allplex™ RP 1/2/3 assays, using BioFire^®^ RP 2.1*plus* panel as the reference assay. The PowerChek™ RVP assay detected HRV and HEV collectively (HRV/HEV), RSV as a single entity (RSV A/B), and influenza A subtypes (H1-2009, H3, NS) as a single Flu A target. Both PowerChek™ RVP and Allplex™ RP detected HBoV, which is not included in the BioFire^®^ RP 2.1*plus* panel; therefore, HBoV comparison was limited to these two assays.

For SARS-CoV-2, PowerChek™ RVP detected both N and ORF1ab genes. Its results were compared only with BioFire^®^ RP 2.1*plus*, as Allplex™ RP does not include SARS-CoV-2. However, due to regulatory restrictions on cloud server locations in the Republic of Korea, Ct values could not be fully retrieved through the Fireworks system, limiting complete data access. CoV-HKU1 was excluded from comparative analysis because it was detected in only one system.

The comparison of the PowerChek™ RVP with the BioFire^®^ RP 2.1*plus* and the Allplex™ RP for detecting respiratory viruses in NPS specimens is summarized in Table 2. The PowerChek™ RVP identified 226 positive cases with six discrepancies, totaling 232 cases. The BioFire^®^ RP 2.1*plus* identified 224 positive cases, while the Allplex™ RP detected 188 positive cases with 14 discrepancies. PowerChek™ RVP showed high concordance with BioFire^®^ RP 2.1*plus* across most targets, detecting nearly all viruses with minimal discrepancies. Slight variations were observed for specific targets, including AdV (19 positives by PowerChek™ RVP vs. 20 positives by BioFire^®^ RP 2.1*plus*) and influenza B (19 vs. 20).

Compared to Allplex™ RP, PowerChek™ RVP showed improved detection for certain viruses, including hMPV (10 vs. 8), parainfluenza viruses (PIV1, PIV2, and PIV4), and CoV-NL63. Among the 104 negative results identified by BioFire^®^ RP 2.1*plus*, both PowerChek™ RVP and Allplex™ panels produced false-positive results. PowerChek™ RVP yielded 12 false positives. These included two for AdVs, one for PIV1, one for PIV4, and eight for HRV/HEV. Allplex RP yielded three false positives. These included one for HEV, one for HRV, and one for HBoV.

### 3.2. Diagnostic Performance of the Powerchek™ RVP 1/2/3/4 Assay

Diagnostic performance metrics of the PowerChek™ RVP and Allplex™ RP were calculated relative to BioFire^®^ RP 2.1*plus* (Table 3 and Table 4).

For the PowerChek™ RVP, the overall relative sensitivity, specificity, accuracy, PPV, and NPV were 97.4%, 88.5%, 94.6%, 95.0%, and 93.9%, respectively. The relative sensitivity exceeded 95% for most targets except for CoV-NL63 (90.0%), influenza A (90.9%), and PIV3 (90.0%). The relative specificity was 100% for all targets, except AdV (98.1%), PIV1 (99.0%), PIV4 (99.0%), and HRV/HEV (92.3%). The relative accuracy was >90% for all the viruses, with the lowest value observed for HRV/HEV (94.0%).

For the Allplex RP assay, the overall relative sensitivity, specificity, accuracy, PPV, and NPV were 93.1%, 97.1%, 94.4%, 98.4%, and 87.8%, respectively. The relative sensitivities were 100% for most viruses, but lower for CoV-NL63 (80.0%), hMPV (80.0%), PIV1 (88.9%), PIV2 (90.0%), PIV3 (90.0%), PIV4 (80.0%), and Flu A NS (0%). The relative specificity was 100% for all targets except HRV (99.0%) and HEV (99.0%). The relative accuracy remained above 95% for most viruses, with the lowest accuracy observed for influenza A, NS (98.1%) (Table 3).

Cohen’s kappa agreement between the PowerChek™ RVP and BioFire^®^ RP 2.1*plus* ranged from 0.843 to 1.000, indicating excellent agreement for all viral targets. Although the overall kappa values for the PowerChek™ RVP were slightly lower than those for the Allplex™ RP (0.879 to 1.000), this difference was primarily due to the lower agreement observed for HRV and HEV. When HRV/HEV were excluded from the analysis, the overall kappa value increased to 0.955 (0.919 to 0.991) for PowerChek™ and 0.894 (0.841 to 0.948) for Allplex™, indicating a higher agreement for the PowerChek™ panel (Table 4).

### 3.3. Further Analysis for Discrepant Samples

For the 15 discordant specimens that were positive by BioFire^®^ RP 2.1*plus* panel but negative by PowerChek™ RVP or Allplex™ RP, BioFire^®^ RP 2.1*plus* testing was repeated for confirmation, and the results were consistent with the initial findings. Sequencing was performed on these specimens to further investigate these discrepancies (Table 5). Viruses showing false-negative results relative to BioFire^®^ RP 2.1*plus* included AdV, CoV-NL63, Flu B, hMPV PIV1–PIV4, Flu A, and RSV. Eleven discordant results between multiplex respiratory panel assays remained unidentified owing to amplification failure by sequencing. Most patients showed high Ct values (typically >30), indicating low viral load. For the four samples with successful sequencing results, all were concordant with BioFire^®^ RP 2.1*plus* and PowerChek™ RVP results, while Allplex™ RP showed false-negative results. In addition, IC results for both PowerChek™ RVP and Allplex™ RP assays have been included in Supplementary Table S1, and the individual IC values for all 15 discordant specimens were also added to provide additional analytical context.

Additional experiments were conducted to investigate the HRV/HEV-positive results observed in the PowerChek™ RVP or Allplex™ RP assays but not detected by the BioFire^®^ RP 2.1*plus*. As most of these samples exhibited high Ct values, indicative of low viral loads, sequencing was not informative. Therefore, comparative testing was performed using serial dilutions of RNA from patients positive for HEV or HRV, as well as the corresponding reference materials. In patient-specimen testing at a 10^−5^ dilution, Allplex™ RP yielded negative results in two of two samples for both HRV and HEV, whereas PowerChek™ RVP was negative in only one HRV sample while detecting all others (Supplementary Table S2). In reference material testing, Allplex™ RP failed to detect HEV at the lowest tested concentration. HRV became negative at a 1:100 dilution. In contrast, PowerChek™ RVP was detected under these conditions (Supplementary Table S3).

## 4. Discussion

In this study, we evaluated the performance of the PowerChek™ RVP 1/2/3/4 for the detection of 16 respiratory viruses and compared it with the BioFire^®^ RP 2.1*plus* and the Allplex™ RP 1/2/3. The results demonstrated that the PowerChek™ RVP exhibited excellent diagnostic performance for most viruses when BioFire^®^ RP 2.1*plus* was used as the reference standard. The overall relative sensitivity, specificity, and accuracy of PowerChek™ RVP were 97.4%, 88.5%, and 94.6%, respectively. These metrics were comparable to or exceeded those reported in previous studies that used multiplex RT-PCR assays [13,15,16]. Cohen’s Kappa coefficients between the PowerChek™ RVP and BioFire^®^ RP 2.1*plus* panel ranged from 0.843 to 1.000, indicating near-perfect agreement. This suggests that the PowerChek™ RVP is a reliable diagnostic tool, comparable to the widely used BioFire^®^ RP 2.1*plus* panel.

Currently, various commercial multiplex rRT-PCR assays are available for the simultaneous detection of respiratory viruses, including SARS-CoV-2. Most of these assays have been developed by incorporating SARS-CoV-2 targets into existing panels for other common respiratory pathogens, such as influenza and RSV. Recently, several manufacturers have introduced rapid point-of-care (PoC) molecular assays capable of simultaneously detecting influenza A, influenza B, RSV, and SARS-CoV-2 using RT-PCR. Examples include the STANDARD™ M10 Flu/RSV/SARS-CoV-2 test (SD Biosensor Inc., Seoul, Republic of Korea), Xpert^®^ Xpress SARS-CoV-2/Flu/RSV (Cepheid, Sunnyvale, CA, USA), and Savanna^®^ Respiratory Viral Panel-4 (QuidelOrtho, San Diego, CA, USA) [17]. Recent comparative studies have highlighted performance differences among these newly developed rapid multiplex assays. Jensen et al. [18] directly compared the Xpert^®^ Xpress CoV-2/Flu/RSV *plus* and STANDARD™ M10 Flu/RSV/SARS-CoV-2 assays, demonstrating that both platforms provided accurate results for SARS-CoV-2, influenza A/B, and RSV, with the Xpert^®^ assay showing slightly higher analytical and clinical performance. Similarly, the STANDARD™ M10 assay demonstrated excellent concordance with the Xpert^®^ Xpress system for the detection of SARS-CoV-2, influenza A/B, and RSV [19]. Collectively, these studies highlight the comparable diagnostic reliability of rapid molecular platforms in clinical use.

In this study, Allplex™ RP assays and BioFire^®^ RP 2.1*plus* were used for comparison. The STANDARD™ M10 assay was directly compared with the Allplex™ assay, showing an overall agreement of 99.5% [20]. Similarly, in a comparative evaluation between the Allplex™ and BioFire^®^ FilmArray panels, high concordance was observed for most respiratory viral targets except for HEV/HRV. Although, as far as we know, there has been no study that directly compared the PowerChek™ assay with the recently developed Xpert^®^ Xpress or STANDARD™ M10 systems. This present study, combined with previous comparisons involving Allplex™, suggests that PowerChek™ exhibits a comparable performance level among currently available multiplex molecular assays.

The PowerChek™ RVP 1/2/3/4 assay has several advantages. This was the first multiplex RT-PCR panel in the Republic of Korea to include SARS-CoV-2, enabling the comprehensive screening of 16 respiratory viruses. In this study, the assay successfully detected all 30 SARS-CoV-2 positive cases, showing complete concordance with the BioFire^®^ RP 2.1*plus* panel. With a relatively short runtime and high-throughput capacity, it is suitable for diverse clinical settings. The assay design, based on updated Global Initiative on Sharing All Influenza Data and National Center for Biotechnology Information data, includes internal human gene controls and TaqMan^®^ probe-based fluorescence detection, ensuring accuracy and reducing signal interference. It is available in both strip and tube formats, thereby supporting the flexibility of laboratories with varying capacities. Although it requires a separate nucleic acid extraction step, its four-tube multiplex format streamlined workflow allows for the efficient detection of multiple viral targets, making it a practical and scalable diagnostic solution.

When comparing TAT performance, the BioFire system demonstrated the shortest TAT for single-sample testing. However, for three or more specimens, the 96-well plate–based assays from Kogen and Seegene kits were markedly more efficient in TAT. Although the PowerChek™ RVP assay accommodates a smaller number of samples per batch compared with the Allplex™ RP assay, its PCR amplification time is approximately 40 min shorter. Consequently, when processing between 4 and 22 samples, the PowerChek™ RVP exhibited the most favorable TAT (Table 1). In addition, the PowerChek™ RVP was found to be more cost-effective, highlighting its advantages in both operational efficiency and economic feasibility.

Since the COVID-19 pandemic, the seasonal patterns of respiratory viruses in children have shifted [21,22], highlighting the need for multiplex PCR panels that can detect SARS-CoV-2 as well as other respiratory viruses. PowerChek™ RVP can cover a broad range of viruses and can enable comprehensive and timely clinical responses.

Despite its strong performance, a few discrepancies were observed between the PowerChek™ RVP and comparator assays. PowerChek™ RVP showed higher concordance with BioFire^®^ RP 2.1*plus* than Allplex™ RP across the 15 discordant samples. These differences may have been influenced by the study design, as sequencing was performed on the residual samples after all three assays. In some cases, the sequencing results were interpreted as unidentified, which may not reflect the true absence of the virus. Certain discordant results, particularly for AdV, hMPV, PIV, and HRV/HEV, remain inconclusive even after sequencing. These findings align with those of previous studies reporting high concordance between multiplex PCR assays and sequencing, while also suggesting that occasional false positive or negative results may be attributed to factors such as low viral load or target cross-reactivity [16,23,24]. Moreover, differences in nucleic acid extraction methods may also have affected the results, particularly in samples with low viral loads [25]. Since all three assays used a magnetic bead-based extraction method, any observed differences in performance, especially in low-titer specimens, are attributable to the specific extraction platform. Unlike PowerChek™ RVP and Allplex™ RP, which require manual transfer of nucleic acids, the BioFire^®^ RP 2.1*plus* employs an integrated sample-to-answer cartridge system. This workflow difference might affect detection sensitivity in low viral load samples.

In addition to cross-reactivity and low viral loads, differences in the limit of detection between the kits and viral targets may also contribute to the assay discrepancies. Previous reports have shown that, even within the same multiplex assay, the lowest detectable concentration can vary substantially depending on the virus [26]. These differences should be interpreted with caution, as a higher positivity for certain viruses may reflect a lower limit of detection than false-positive results. Additionally, negative specimens were obtained from symptomatic patients, suggesting that some viruses may have been present below the detection limits of the assays. In this study, according to the manufacturer’s interpretation guidelines [27], Flu A, No Subtype (NS) results are defined as cases in which the Influenza A pan assay is positive but all subtype-specific assays are negative. In addition, Kogene conducted in silico analysis using Global Initiative on Sharing All Influenza Data (GISAID) from 1 June 2025 to 21 October 2025 and demonstrated 100% predicted detection of circulating influenza A strains. Similarly, routine in silico monitoring using GISAID between June 2023 and June 2024 showed that more than 99% of circulating influenza A strains are predicted to be detected by the BioFire^®^ RP 2.1*plus* panel [28], supporting the stable performance of both platforms.

Notably, specificity for HRV/HEV was 92.3%, the lowest among 16 viral targets in the PowerChek™ RVP panel. The overall accuracy of this analyte was the lowest. Eight false-positive cases were observed in PowerChek™ RVP despite being negative in the BioFire^®^ RP 2.1*plus* assay. Previous studies reported that the Allplex™ RP detected more HEV and HRV cases than those of BioFire^®^ RP. Most of these additional positives were associated with high Ct values, suggesting superior analytical sensitivity of the Allplex™ assays for these targets [29].

For HRV/HEV the supplementary experiments demonstrated higher analytical sensitivity of the PowerChek™ RVP assay compared to the Allplex™ RP. However, due to the use of water as the diluent, ICs (GAPDH for PowerChek™ RVP and MS2 phage for Allplex™ RP) were not consistently detected at higher dilutions, particularly near the limit of detection (Supplementary Table S2). While such results would be classified as invalid in clinical diagnostics, they are appropriate in the context of analytical sensitivity testing, as they reflect the assay’s detection capability rather than procedural failure. The IC signal was absent in the Seegene assay when using commercial RNA reference material. This observation is consistent with the omission of the nucleic acid extraction process, during which the IC (MS2 phage) is normally incorporated, thereby explaining the lack of IC detection (Supplementary Table S3).

Overall, sequencing is essential for resolving inconsistencies, while PowerChek™ RVP exhibits reliable diagnostic potential comparable to BioFire^®^ RP 2.1*plus* and Allplex™ RP [15,16]. Future studies should investigate analytical sensitivity and causes of discordant results in greater depth.

This study has some limitations. First, this was a single-center study with a relatively small sample size, which may have limited the generalizability of the findings. In particular, regional variations in circulating strains could influence detection performances. Thus, the results may not fully represent regional generalizability. Second, the sample size for certain low-prevalence viruses like CoV-229E was relatively small, which can reduce the statistical power of this study. Third, HBoV comparison could not be performed against BioFire^®^ RP 2.1*plus*, limiting the evaluation of its diagnostic contribution. Finally, some discordant cases remained unresolved even after sequencing, particularly for CoV, IFV, hMPV, and PIV, suggesting that low viral loads or cross-reactivity may continue to challenge multiplex assays.

## 5. Conclusions

The PowerChek™ RVP 1/2/3/4 assay demonstrated excellent diagnostic performance, with high sensitivity, specificity, and near-perfect concordance with the BioFire RP 2.1*plus*. These findings highlight the complementary value of multiple diagnostic platforms and emphasize the need for confirmatory methods such as sequencing to resolve discordant results. Given its high throughput and cost-effectiveness, the PowerChek™ RVP panel is particularly well suited for large-scale syndromic screening in clinical laboratories and public health settings. Future multicenter studies with larger sample sizes and detailed cost-effectiveness analyses are warranted to validate the clinical utility of the PowerChek™ RVP panel.

## Figures and Tables

**Table 1:** Specifications of three kinds of real-time PCR for respiratory virus detection.

Specification	PowerChek™ RVP (Kogene, Seoul, Republic of Korea)	Allplex™ RP (Seegene, Seoul, Republic of Korea)	Biofire^®^ RP 2.1*plus* (BioMérieux, Salt Lake City, UT, USA)
Main detection principle	Multiplex Real-time RT-PCR (TaqMan probe)	Multiplex Real-time RT-PCR (MuDT™)	Nested multiplex PCR with syndromic panel
Number of virus targets	16	19	19
Test number in one run	22 specimens can be processed per run	30 specimens can be processed per run	Single cartridge per test; 23 targets per test
Turnaround time (TAT)	1 h 30 min	2 h 10 min	50 min
Primer information	Target-specific primers for key respiratory viruses	Multiplex primer sets for multiple viral targets	Preloaded primers in a closed system
Estimated cost per test	USD 30~40	USD 40~50	>USD 200 (Higher owing to single-use cartridge)
Hands-on time	40min: Sample prep and RNA extraction	40min: Sample prep and RNA extraction	5 min
PCR run time	100 min	150 min	45 min
Batch throughput	22 samples	30 samples	N/A
Internal control (IC)	GAPDH	Bacteriophage MS2	RNA process control (*Schizosaccharomyces pombe*) Array PCR control
Others	Detection of 16 targets, including SARS-CoV-2 Pre-Mix type	Automated workflow available	Fully automated, minimal hands-on time, individually testable

Abbreviations: GAPDH; Glyceraldehyde 3-phosphate dehydrogenase, N/A; Not applicable.

**Table 2:** Comparison of the PowerChek™ RVP 1/2/3/4 with the Biofire^®^ RP 2.1*plus* and the Allplex™ RP 1/2/3 assays for the detection of respiratory viruses in NPS specimens.

BioFire^®^ RP 2.1*plus* (BioMérieux)			Allplex™ RP (Seegene)			PowerChek™ RVP (Kogene)			Total
Target	Positive	Negative	Target	Positive	Negative	Target	Positive	Negative	
AdV	20	0	AdV	20	0	AdV	19	1	20
CoV-229E	3	0	CoV-229E	3	0	CoV-229E	3	0	3
CoV-NL63	10	0	CoV-NL63	8	2	CoV-NL63	9	1	10
CoV-OC43	10	0	CoV-OC43	10	0	CoV-OC43	10	0	10
Flu A-H3	10	0	Flu A-H3	10	0	Flu A	20	2	10
FluA-H1-2009	10	0	Flu A-H1-2009	10	0				10
Flu A, NS	2	0	Flu A, NS	0	2				2
Flu B	20	0	Flu B	18	2	Flu B	19	1	20
hMPV	10	0	hMPV	8	2	hMPV	10	0	10
PIV1	9	0	PIV1	8	1	PIV1	9	0	9
PIV2	10	0	PIV2	9	1	PIV2	10	0	10
PIV3	10	0	PIV3	9	1	PIV3	9	1	10
PIV4	10	0	PIV4	8	2	PIV4	10	0	10
HBoV	-	-	HBoV	8	0	HBoV	8	0	8
RSV	30	0	RSV A	15	0	RSV	30	0	30
			RSV B	14	1				
HRV/HEV	30	0	HRV	15	0	HRV/HEV	30	0	30
			HEV	15	0				
SARS-CoV-2	30	0	SARS-CoV-2	-	-	SARS-CoV-2	30	0	30
Total No.	224	0		188	14		226	6	232

BioFire^®^ RP 2.1*plus* does not include HBoV. Allplex™ RP 1/2/3 does not include SARS-CoV-2. PowerChek™ RVP panels detect RSV and HRV/HEV as combined targets. Allplex™ RP detect RSV A/B and HRV/HEV separately. In the table, the results for these viruses are aggregated across panels for comparison. “-”: Data not available. Abbreviations: AdV: Adenovirus; CoV: Coronavirus; Flu A: Influenza virus A; Flu B: Influenza virus B; hMPV: Human Metapneumovirus; PIV: Parainfluenza virus; HBoV: Human Bocavirus; RSV: Respiratory Syncytial Virus; HRV: Human Rhinovirus; HEV: Human Enterovirus; SARS-CoV-2: Severe Acute Respiratory Syndrome Coronavirus 2.

**Table 3:** Diagnostic performance of the PowerChek™ RVP 1/2/3/4 and Allplex™ RP 1/2/3 for respiratory viral detection, using the BioFire^®^ RP 2.1*plus* as the reference assay.

Pathogen	Relative Sensitivity		Relative Specificity		Relative Accuracy (*)	
Target	PowerChek™ RVP	Allplex™ RP	PowerChek™ RVP	Allplex™ RP	PowerChek™ RVP	Allplex™ RP
AdV	95.0%	100%	98.1%	100%	97.6%	100%
CoV-229E	100%	100%	100%	100%	100%	100%
CoV-NL63	90%	80.0%	100%	100%	99.1%	98.2%
CoV-OC43	100%	100%	100%	100%	100%	100%
Flu A-H3	90.9%	100%	100%	100%	98.4%	100%
FluA-H1-2009		100%		100%		100%
Flu A, NS		0%		100%		98.1%
Flu B	95.0%	90.0%	100%	100%	99.2%	98.4%
hMPV	100%	80.0%	100%	100%	100%	98.2%
PIV1	100%	88.9%	99.0%	100%	99.1%	99.1%
PIV2	100%	90.0%	100%	100%	100%	99.1%
PIV3	90.0%	90.0%	100%	100%	99.1%	99.1%
PIV4	100%	80.0%	99.0%	100%	99.1%	98.2%
HBoV	-	-	-	-	-	-
RSV A	100%	100%	100%	100%	100%	100%
RSV B		93.3%		100%		99.2%
HRV	100%	100%	92.3%	99.0%	94.0%	99.2%
HEV		100%		99.0%		99.2%
SARS-CoV-2	100%	-	100%	-	100%	-
Overall	97.4%	93.1%	88.5%	97.1%	94.6%	94.4%

Biofire^®^ RP 2.1*plus* does not include HBoV. Allplex™ RP 1/2/3 does not include SARS-CoV-2. PowerChek™ RVP panels detect RSV and HRV/HEV as combined targets. Allplex™ RP detect RSV A/B and HRV/HEV separately. In the table, the results for these viruses are aggregated across panels for comparison. (*) These values are dependent on disease prevalence; “-”: Data not available. Abbreviations: AdV: Adenovirus; CoV: Coronavirus; Flu A: Influenza virus A; Flu B: Influenza virus B; hMPV: Human Metapneumovirus; PIV: Parainfluenza virus; HBoV: Human Bocavirus; RSV: Respiratory Syncytial Virus; HRV: Human Rhinovirus; HEV: Human Enterovirus; SARS-CoV-2: Severe Acute Respiratory Syndrome Coronavirus 2.

**Table 4:** Agreement of the PowerChek™ RVP 1/2/3/4 and Allplex™ RP 1/2/3 with the BioFire^®^ RP 2.1*plus* for respiratory viral detection.

Pathogen	BioFire^®^ RP 2.1*plus* vs. PowerChek™ RVP		BioFire^®^ RP 2.1*plus* vs. Allplex™ RP	
	Kappa	95% C.I.	Kappa	95% C.I.
AdV	0.912	0.815–1.000	1.000	1.000–1.000
CoV-229E	1.000	1.000–1.000	1.000	1.000–1.000
CoV-NL63	0.943	0.831–1.000	0.879	0.715–1.000
CoV-OC43	1.000	1.000–1.000	1.000	1.000–1.000
Flu A-H3	0.943	0.864–1.000	1.000	1.000–1.000
FluA-H1-2009			1.000	1.000–1.000
Flu A, NS			0	0–0
Flu B	0.970	0.910–1.000	0.938	0.853–1.000
hMPV	1.000	1.000–1.000	0.879	0.715–1.000
PIV1	0.943	0.831–1.000	0.936	0.813–1.000
PIV2	1.000	1.000–1.000	0.943	0.831–1.000
PIV3	0.943	0.831–1.000	0.943	0.831–1.000
PIV4	0.948	0.845–1.000	0.879	0.715–1.000
HBoV	-	-	-	-
RSV A	1.000	1.000–1.000	1.000	1.000–1.000
RSV B			0.961	0.884–1.000
HRV	0.843	0.739–0.947	0.963	0.891–1.000
HEV			0.963	0.891–1.000
SARS-CoV-2	1.000	1.000–1.000	-	-
Overall	0.873	0.816–0.930	0.879	0.824–0.935

BioFire^®^ RP 2.1*plus* does not include HBoV. Allplex™ RP 1/2/3 does not include SARS-CoV-2. PowerChek™ RVP panels detect RSV and HRV/HEV as combined targets. Allplex™ RP detect RSV A/B and HRV/HEV separately. In the table, the results for these viruses are aggregated across panels for comparison. “-”: Data not available. Abbreviations: C.I.: Confidence Interval; AdV: Adenovirus; CoV: Coronavirus; Flu A: Influenza virus A; Flu B: Influenza virus B; hMPV: Human Metapneumovirus; PIV: Parainfluenza virus; HBoV: Human Bocavirus; RSV: Respiratory Syncytial Virus; HRV: Human Rhinovirus; HEV: Human Enterovirus; SARS-CoV-2: Severe Acute Respiratory Syndrome Coronavirus 2.

**Table 5:** Comparison of discordant results among PowerChek™ RVP, Allplex™ RP, and BioFire^®^ RP 2.1*plus* with sequencing data.

Specimen No.	Target Virus	BioFire^®^RP 2.1*plus*	PowerChek™ RVP			Allplex™ RP			Virus Sequencing		Interpretation
		Results	Results	Ct	IC (Ct)	Results	Ct	IC (Ct)	Target	Result	
8	AdV	AdV	Neg	-	21.31	AdV	37.4	28.74	AdV	N/A	Unidentified
28	CoV-NL63	CoV-NL63	CoV-NL63	31.19	19.74	Neg	-	29.85	CoV-NL63	N/A	Unidentified
29	CoV-NL63	CoV-NL63	Neg	-	20.49	Neg	-	29.48	CoV-NL63	N/A	Unidentified
79	Flu B	Flu B	Neg	-	22.91	Neg	-	28.71	Flu B	N/A	Unidentified
83	Flu B	Flu B	Flu B	31.29	25.22	Neg	-	28.55	Flu B	N/A	Unidentified
90	hMPV	hMPV	hMPV	29.4	20.28	Neg	-	27.96	hMPV	N/A	Unidentified
92	hMPV	hMPV	hMPV	29.2	21.6	Neg	-	30.9	hMPV	hMPV	hMPV
100	PIV1	PIV1	PIV1	18.96	25.75	Neg	-	29.03	PIV1	PIV1	PIV1
103	PIV2	PIV2	PIV2	31.18	21.44	Neg	-	30.32	PIV1	N/A	Unidentified
122	PIV3	PIV3	Neg	-	20.35	Neg	-	29.55	PIV3	N/A	Unidentified
125	PIV4	PIV4	PIV4	26.33	21.34	Neg	-	30.36	PIV4	PIV4	PIV4
132	PIV4	PIV4	PIV4	19.57	23.73	Neg	-	34.46	PIV4	PIV4	PIV4
141	Flu A,NS	Flu A	Neg	-	26.46	Neg	-	32.57	Flu A	N/A	Unidentified
142	Flu A, NS	Flu A	Neg	-	21.06	Neg	-	31	Flu A	N/A	Unidentified
179	RSV	RSV	RSV	32.17	18.58	Neg	-	29.99	RSV	N/A	Unidentified

“-”: Data not available. Abbreviations: IC: Internal control; Ct: Cycle threshold; AdV: Adenovirus; CoV: Coronavirus; Flu A: Influenza virus A; Flu B: Influenza virus B; hMPV: Human Metapneumovirus; PIV: Parainfluenza virus; Neg: Negative, N/A: Not applicable.

## Data Availability

The data from this study are not publicly available due to privacy and/or ethical restrictions.

## Supplementary Materials

The following supporting information can be downloaded at: https://www.mdpi.com/article/10.3390/diagnostics15212713/s1. Table S1: Internal Control (IC) Results for PowerChek™ vs. Allplex™; Table S2: Comparison of PowerChek™ and Allplex™ assays for the detection of HRV/HEV in BioFire^®^-confirmed clinical specimens: discrepancies verified by serial dilution; Table S3: Comparison of PowerChek™ and Allplex™ assay performance for HRV/HEV on reference material: discrepancies verified by serial dilution.

## Abbreviations

The following abbreviations are used in this manuscript:

ARI	acute respiratory infections
AdV	adenovirus
HBoV	human bocavirus
CoV	coronavirus
RSV	respiratory syncytial virus
HEV	human enterovirus
HRV	human rhinovirus
PIV	parainfluenza virus
HMPV	human metapneumovirus
TAT	turnaround time
PCR	polymerase chain reaction
RT-PCR	multiplex reverse transcription PCR
RP	respiratory panel
IFV	influenza viruses
NPS	nasopharyngeal swabs
PPV	positive predictive value
NPV	negative predictive value
Ct	Cycle threshold
RVP	respiratory virus panel
PoC	point-of-care
