# Correction: Lee et al. Evaluation of the PowerChek™ Respiratory Virus Panel 1/2/3/4 for the Detection of 16 Respiratory Viruses: A Comparative Study with the Allplex™ Respiratory Panel Assay 1/2/3 and BioFire^®^ Respiratory Panel 2.1 *plus*. *Diagnostics* 2025, *15*, 2713

**DOI:** 10.3390/diagnostics15243094

**Published:** 2025-12-05

**Authors:** Hyeongyu Lee, Rokeya Akter, Jong-Han Lee, Sook Won Ryu

**Affiliations:** 1 Department of Laboratory Medicine, Wonju College of Medicine, Yonsei University, Wonju 26426, Republic of Korea; sinoull@naver.com (H.L.); akterbinteha30@yonsei.ac.kr (R.A.); 2 Research Institute of Metabolism and Inflammation, Wonju College of Medicine, Yonsei University, Wonju 26426, Republic of Korea; 3 Department of Laboratory Medicine, School of Medicine, Kangwon National University, Chuncheon 24341, Republic of Korea

In the original publication [1], there was a mistake in Table 1 as published. The Turnaround Time (TAT) values were incorrectly written as “PowerChek—1 h 30 min, Allplex—2 h 10 min”. The TAT values should represent the total time required, calculated as the sum of the Hands-on Time and PCR Run Time. Accordingly, the correct values are “PowerChek—2 h 20 min, Allplex—3 h 10 min”. The corrected version of Table 1 appears below. The authors state that the scientific conclusions are unaffected. This correction was approved by the Academic Editor. The original publication has also been updated.

## Figures and Tables

**Table 1:** Specifications of three kinds of real-time PCR for respiratory virus detection.

Specification	PowerChek™ RVP (Kogene, Seoul, Republic of Korea)	Allplex™ RP (Seegene, Seoul, Republic of Korea)	Biofire^®^ RP 2.1*plus* (BioMérieux, Salt Lake City, UT, USA)
Main detection principle	Multiplex Real-time RT-PCR (TaqMan probe)	Multiplex Real-time RT-PCR (MuDT™)	Nested multiplex PCR with syndromic panel
Number of virus targets	16	19	19
Test number in one run	22 specimens can be processed per run	30 specimens can be processed per run	Single cartridge per test; 23 targets per test
Turnaround time (TAT)	2 h 20 min	3 h 10 min	50 min
Primer information	Target-specific primers for key respiratory viruses	Multiplex primer sets for multiple viral targets	Preloaded primers in a closed system
Estimated cost per test	USD 30~40	USD 40~50	>USD 200 (Higher owing to single-use cartridge)
Hands-on time	40 min: Sample prep and RNA extraction	40 min: Sample prep and RNA extraction	5 min
PCR run time	100 min	150 min	45 min
Batch throughput	22 samples	30 samples	N/A
Internal control (IC)	GAPDH	Bacteriophage MS2	RNA process control (*Schizosaccharomyces pombe*) Array PCR control
Others	Detection of 16 targets, including SARS-CoV-2 Pre-Mix type	Automated workflow available	Fully automated, minimal hands-on time, individually testable

Abbreviations: GAPDH; Glyceraldehyde 3-phosphate dehydrogenase, N/A; Not applicable.
