# Supplementary material for: Evaluation of the PowerChek™ Respiratory Virus Panel 1/2/3/4 for the Detection of 16 Respiratory Viruses: A Comparative Study with the Allplex™ Respiratory Panel Assay 1/2/3 and BioFire® Respiratory Panel 2.1 plus
Source: Diagnostics (Basel). 2025 Oct 27;15(21):2713. doi: 10.3390/diagnostics15212713 (PMC12610354; doi:10.3390/diagnostics15212713)
Supplement: Supplementary file 1 [file diagnostics-15-02713-s001.zip › diagnostics-3929690-supplementary.pdf]

Evaluation of the PowerChek™ Respiratory Virus Panel 1/2/3/4 for the Detection of 16 Respiratory Viruses: A Comparative Study with the Allplex™ Respiratory Panel assay 1/2/3 and BioFire® Respiratory Panel 2.1 *plus*

Hyeongyu Lee [1,†], Rokeya Akter [1,†], Jong-Han Lee [1,2*], Sook Won Ryu [3,*]

Supplementary Table S1. Internal Control (IC) Results for PowerChek™ vs Allplex™

All clinical specimens (N)	PowerChek™ RVP		Allplex™ RP	
	IC Ct median	IC Ct Range	IC Ct median	IC Ct Range
336	21.23	13.1~29.36	26.83	17.23~36.42

Abbreviations: Ct: Cycle Threshold; IC: Internal control.

Supplementary Table S2. Comparison of PowerChek™ and Allplex™ assays for the detection of HRV/HEV in biofire®-confirmed clinical specimens: discrepancies verified by serial dilution

Specimen no.	Target Virus	BIOFIRE® RP2.1 *plus*	Tenfold serial dilutions	PowerChek™ RVP			Allplex™ RP		
		Results	(10^{-x})	Results	Ct value	IC (Ct)	Results	Ct value	IC (Ct)
1	HEV	HRV/HEV	10^{-2}	HEV	22.25	25.01	HEV	30.62	34.53
				HEV	22.24	25.07	HEV	30.24	34.19
			10^{-3}	HEV	25.52	28.53	HEV	33.87	N/A
				HEV	25.48	28.35	HEV	33.58	N/A

			10^{-4}	HEV	28.34	30.95	HEV	36.83	N/A
				HEV	28.16	31.9	HEV	36.7	N/A
			10^{-5}	HEV	31.14	N/A	Neg	N/A	N/A
				HEV	30.07	33.38	Neg	N/A	N/A
2	HEV	HRV/HEV	10^{-3}	HEV	22.35	29.14	HEV	31.33	N/A
				HEV	22.27	29.15	HEV	31.31	38.8
			10^{-4}	HEV	25.8	36.57	HEV	34.87	N/A
				HEV	25.55	31.41	HEV	34.43	N/A
			10^{-5}	HEV	28.51	N/A	HEV	37.82	N/A
				HEV	28.4	N/A	HEV	38.06	N/A
			10^{-6}	HEV	30.7	N/A	Neg	N/A	N/A
				HEV	30.01	N/A	HEV	40.56	N/A
3	HRV	HRV/HEV	10^{-3}	HRV	24.97	27.05	HRV	32.19	41.24
				HRV	25.07	27.12	HRV	32.2	40.72
			10^{-4}	HRV	27.67	30.52	HRV	35.94	N/A
				HRV	27.88	30.46	HRV	36.13	N/A
			10^{-5}	HRV	30.4	35.25	HRV	38.94	N/A
				HRV	30.04	31.86	HRV	40	N/A
			10^{-6}	HRV	31.83	33.35	HRV	40.47	N/A
				HRV	32.55	N/A	Neg	N/A	N/A
4	HRV	HRV/HEV	10^{-3}	HRV	23.62	26.55	HRV	32.84	40.72
				HRV	23.63	26.42	HRV	32.65	41.06
			10^{-4}	HRV	26.48	29.47	HRV	36.24	N/A
				HRV	27.09	29.19	HRV	35.65	N/A

10^{-5}	HRV	29.28	N/A	HRV	39.08	N/A
	HRV	29.8	N/A	HRV	39.36	N/A
10^{-6}	HRV	31.73	N/A	Neg	N/A	N/A
	Neg	N/A	35.83	Neg	N/A	N/A

"-": Data not available. **Abbreviations**: Ct: Cycle Threshold; HRV: Human Rhinovirus; HEV: Human Enterovirus; Neg: Negative, N/A: Not applicable

Supplementary Table S3. Comparison of PowerChek™ and Allplex™ assay performance for HRV/HEV on reference material: discrepancies verified by serial dilution

Reference Material ID	Target Virus	Titer	PowerChek™ RVP			Allplex™ RP		
		(Unit)	Results	Ct value	IC (Ct)	Results	Ct value	IC (Ct)
Human Coxsackievirus A6 (ATCC VR-1801)	HEV	8.9×10^4 ($TCID_{50}$ /mL)	HEV	18.02	25.46	HEV	29.01	N/A
			HEV	17.89	25.45	HEV	28.9	N/A
			HEV	18.07	25.77	HEV	28.79	N/A
		8.9×10^3 ($TCID_{50}$ /mL)	HEV	21.17	28.93	HEV	31.88	N/A
			HEV	21	28.41	HEV	31.95	N/A
			HEV	21.02	28.71	HEV	32.06	N/A
		8.9×10^2 ($TCID_{50}$ /mL)	HEV	24.25	32.05	HEV	36.33	N/A
			HEV	24.3	32.15	HEV	35.38	N/A
			HEV	24.12	31.04	HEV	36.51	N/A
		8.9×10^1	HEV	27.2	N/A	Neg	N/A	N/A

		($TCID_{50}$ /mL)	HEV	27.46	34.91	Neg	N/A	N/A
			HEV	27.04	34.97	Neg	N/A	N/A
Human Rhinovirus 14 (KBPV-VR-39D)	HRV	2.0 × 10^4 (PFU/mL)	HRV	18.35	32.09	HRV	33.25	N/A
			HRV	18.15	30.49	HRV	33.19	N/A
			HRV	18.22	31.15	HRV	33.29	N/A
		2.0 × 10^3 (PFU/mL)	HRV	21.12	37.91	HRV	36.89	N/A
			HRV	21.57	N/A	HRV	37.64	N/A
			HRV	21.55	36.45	HRV	36.97	N/A
		2.0 × 10^2 (PFU/mL)	HRV	24.81	N/A	Neg	N/A	N/A
			HRV	24.74	N/A	Neg	N/A	N/A
			HRV	24.53	N/A	Neg	N/A	N/A
		2.0 × 10^1 (PFU/mL)	HRV	27.19	N/A	Neg	N/A	N/A
			HRV	27.61	N/A	Neg	N/A	N/A
			HRV	27.54	N/A	Neg	N/A	N/A
		2.0 × 10^0 (PFU/mL)	HRV	30.24	N/A	Neg	N/A	N/A
			HRV	30.13	N/A	Neg	N/A	N/A
			HRV	30.92	N/A	Neg	N/A	N/A

"-": Data not available. **Abbreviations**: Ct; Cycle Threshold; $TCID_{50}$/mL: 50% Tissue Culture Infection Dose/mL; PFU/mL: Plaque-Forming Units/mL; HRV: Human Rhinovirus; HEV: Human Enterovirus; Neg: Negative, N/A: Not applicable; ATCC: American Type Culture Collection; KBPV: Korea Bank for Pathogenic Viruses
